# Principles of digital professionalism for the metaverse in healthcare

**DOI:** 10.1186/s12911-024-02607-y

**Published:** 2024-07-22

**Authors:** Zahra Mohammadzadeh, Mehdi Shokri, Hamid Reza Saeidnia, Marcin Kozak, Agostino Marengo, Brady D Lund, Marcel Ausloos, Nasrin Ghiasi

**Affiliations:** 1 Department of Health Information Management and Technology, Kashan University of Medical Sciences, Kashan, Iran; 2 Health Information Management Research Center, Kashan University of Medical Sciences, Kashan, Iran; 3 Department of Pediatrics, School of Medicine Emam Khomeini Hospital, Ilam University of Medical Sciences, Ilam, Iran; 4 Department of Knowledge and Information Science, Tarbiat Modares University, (TMU), Tehran, Iran; 5 University of Information Technology and Management in Rzeszow, Rzeszow, 35-225 Poland; 6 Department of Human Science, University of Foggia, Foggia, 71122 Italy; 7 Department of Information Science, University of North Texas, Denton, TX 76203 USA; 8 School of Business, University of Leicester, Leicester, LE2 1RQ UK; 9 Department of Statistics and Econometrics, Bucharest University of Economic Studies, Bucharest, 010552 Romania; 10 Department of Public Health, School of Health, Ilam University of Medical Sciences, Ilam, Iran

**Keywords:** Metaverse, Virtual reality, Digital Professionalism, Healthcare

## Abstract

**Background:**

Experts are currently investigating the potential applications of the metaverse in healthcare. The metaverse, a groundbreaking concept that arose in the early 21st century through the fusion of virtual reality and augmented reality technologies, holds promise for transforming healthcare delivery. Alongside its implementation, the issue of digital professionalism in healthcare must be addressed. Digital professionalism refers to the knowledge and skills required by healthcare specialists to navigate digital technologies effectively and ethically. This study aims to identify the core principles of digital professionalism for the use of metaverse in healthcare.

**Method:**

This study utilized a qualitative design and collected data through semi-structured online interviews with 20 medical information and health informatics specialists from various countries (USA, UK, Sweden, Netherlands, Poland, Romania, Italy, Iran). Data analysis was conducted using the open coding method, wherein concepts (codes) related to the themes of digital professionalism for the metaverse in healthcare were assigned to the data. The analysis was performed using the MAXQDA software (VER BI GmbH, Berlin, Germany).

**Results:**

The study revealed ten fundamental principles of digital professionalism for the metaverse in healthcare: Privacy and Security, Informed Consent, Trust and Integrity, Accessibility and Inclusion, Professional Boundaries, Evidence-Based Practice, Continuous Education and Training, Collaboration and Interoperability, Feedback and Improvement, and Regulatory Compliance.

**Conclusion:**

As the metaverse continues to expand and integrate itself into various industries, including healthcare, it becomes vital to establish principles of digital professionalism to ensure ethical and responsible practices. Healthcare professionals can uphold these principles to maintain ethical standards, safeguard patient privacy, and deliver effective care within the metaverse.

**Supplementary Information:**

The online version contains supplementary material available at 10.1186/s12911-024-02607-y.

## Introduction

The metaverse is a hypothetical network of interconnected virtual worlds facilitated by virtual reality and augmented reality headsets [1]. In simple terms, the Metaverse can be described as an infinite, universal, and immersive virtual world that resembles our physical reality but with different environments, objects, and characters [2]. Mystakidis explains that “*the Metaverse is a post-reality universe*,* a perpetual and persistent multiuser environment merging physical reality with digital virtuality*” [1]. The metaverse is based on the convergence of new technologies such as virtual reality (VR) and augmented reality (AR) [1, 2]. Despite being such a rich convergence of existing technologies, the metaverse itself is not considered a new technology. For this very reason, we do not capitalize the word, just like we do not capitalize virtual reality—despite Neal Stephenson in his novel *Snow Crash*, in which he created the term the Metaverse (with a capital “M”).

According to the futurist Bernard Marr, the convergence of three major technological trends is crucial for the practical implementation of the metaverse: telepresence, digital twinning, and blockchain. Telepresence is the aspect of the metaverse that allows an individual to feel as though they are present in some place other than where they are physically located. Digital twinning is a concept within the metaverse that refers to the quality of virtual objects mimicking physical ones. Blockchain, in the context of the metaverse, refers to a digital ledger to track transactions or exchanges of data. These technologies will enable the metaverse to have a great potential impact on healthcare [3]. In combining these three concepts, new ways to deliver healthcare can be developed, which can help treat patients at lower costs [3, 4].

In the context of medicine, the metaverse can be defined as an immersive and interconnected digital universe that encompasses augmented and virtual reality environments, allowing healthcare professionals and patients to engage in virtual interactions and experiences [5]. The metaverse goes beyond the boundaries of individual virtual platforms or worlds, providing a shared space where medical education, research, and healthcare delivery can be revolutionized [6]. The metaverse holds immense potential for transforming healthcare by enabling telemedicine advancements, immersive medical education and training, virtual healthcare environments, collaborative research and development, mental health treatments, and remote monitoring [7]. It offers a virtual realm where healthcare professionals can provide personalized care, engage in collaborative efforts, and enhance patient experiences, ultimately leading to improved healthcare outcomes [8].

The emergence of the metaverse raises important questions for healthcare services. Can the metaverse assist physicians and healthcare practitioners in their daily tasks? Are there any potential negative impacts of the metaverse that could hinder its implementation in healthcare?

This article explores the concept of digital professionalism and its relevance within the healthcare context of the metaverse, using a series of principles as guides for teaching, learning, and practicing in healthcare professions [1, 4]. Therefore, the primary aim of this study is to answer the following question: What are the principles of digital professionalism that should be considered for the successful implementation of the metaverse in healthcare?

## Material & method

This research study utilized a qualitative design, conducting online semi-structured interviews with a targeted sample of medical information and health informatics specialists (MIS & HIS). The sample size was determined based on data saturation, indicating the point at which no new information emerged from the interviews, signifying theoretical saturation had been achieved [9]. An online interview guide was established for semi-structured interviews based on two categories:

- Internal and external considerations; this means identifying factors inside and outside of digital professionalism. Consider the following examples: Patient privacy, Ethical Use of Virtual Environments, Responsible Use of Technology, Maintaining Professional Boundaries, Collaboration and Teamwork, and Professional Conduct and Communication.
- Early foundations; what is required of digital professionalism in the teaching and learning environment at colleges and universities for healthcare specialists, such as Continuous Learning and Professional Development.

We employed a single inclusion criterion for survey participants, a minimum of five years of hospital experience. However, we acknowledge that working experience alone may not indicate expertise in the field of the metaverse. To ensure that participants possessed the necessary expertise, we considered additional factors such as their professional background, educational qualifications, work experience, and any achievements or special projects related to the metaverse. By incorporating these criteria, we aimed to select individuals who had a strong foundation and demonstrated knowledge in the subject matter of the metaverse. This approach allowed us to increase the likelihood of obtaining valuable insights from participants with expertise in the field. Informed consent was collected from all participants.

The online interviews were conducted in early March 2022, by the first and the second authors of this paper (Z.M. and M.SH). The online interviews were conducted with 20 medical information and health informatics specialists in eight countries (USA, UK, Sweden, Netherlands, Poland, Romania, Italy and Iran) in Zoom Video Communications (See supplementary file 1 conducted interviews). These specialists were recruited through a call on social networks (Instagram, Facebook, and Telegram). The online interview duration ranged from 25 to 55 min. Among the interviewees, eight had over ten years of working experience in hospitals, and about half had a Master’s degree in HIS, seven holding a Ph.D. (Table 1).

**Table 1:** Characteristics of the participants

Participant No.	Gender	Countries	Work experience (years)	Education
1	Female	Iran	6	MSc in HIS
2	Female		8	MSc in MIS
3	Male		9	PhD
4	Male	USA	11	MSc in HIS
5	Male		7	MSc in HIS
6	Female	UK	9	PhD
7	Male		14	MSc in HIS
8	Female		18	PhD
9	Female	Sweden	15	MSc in HIS
10	Male		12	PhD
11	Male		6	MSc in MIS
12	Male	Netherlands	6	MSc in MIS
13	Female		6	MSc in HIS
14	Female		8	MSc in MIS
15	Male	Poland	9	PhD
16	Male		11	MSc in HIS
17	Male	Romania	7	MSc in HIS
18	Female		9	PhD
19	Male	Italy	11	MSc in HIS
20	Male		12	PhD

The online interviews were then transcribed in full for further analysis. Data analysis was performed by the open coding method, a qualitative method in which concepts (codes) are assigned to the data [10]. The analysis was conducted in the MAXQDA software (VER BI GmbH, Berlin, Germany) (One example of conducted analysis was conducted in the MAXQDA software, See supplementary file 2).

## Results

### Main categories

The thematic analysis led to distinguishing two main categories: internal or external considerations, and early foundations. Table 2 depicts these categories, along with their sub-categories and codes. The following sections analyze the categories in detail.

**Table 2:** The main categories, along with their sub-categories and codes

Category with subcategories	Codes
Internal and external considerations	
***Organizational***	*Ethical standards*,* Maintaining Professional Boundaries*,* Maintaining data encryption*,* Cybersecurity measures*,* The integrity of patient data and virtual interactions*,* Informed Consent*,* Confidentiality*,
***Individual***	*Mutual respect*,* Accuracy*,* Discriminatory or offensive behavior*,* Respectful and appropriate language*,* Patient Confidentiality*,* Sharing of expertise*,* Responsible Use of Technology*,* Professional demeanor*,* Respecting autonomy*,* Ethical Use of Virtual Environments*
***Society***	*Collaboration and Teamwork*,* Cultural Competence and Diversity*,* Promoting inclusivity*,* Patient privacy*,* Professional Conduct and Communication*
**Early Foundations**	**Codes**
***Teaching***	*Training*,* Education*,
***Learning***	*Professional Development*,* Stay updated*,* Being aware of potential risks*,* Continuous Learning*

### Internal and external considerations

In the context of digital professionalism for the metaverse in healthcare, the code “internal and external considerations of digital professionalism” emphasized the significance of organizational readiness and identifying organizational risks. These aspects were found to be the most important and effective factors, underscoring the need for healthcare specialists to be well-prepared and capable of recognizing and addressing these considerations proactively.

Participant #2: *“Technological changes and the introduction of new technologies in healthcare require new working methods. Access to and use of digital technology by healthcare providers (i.e.*,* physicians and nurses) lags behind other professions. Maintaining digital professionalism requires organizational readiness to adapt to new technologies”.*

Participant #3: *“Given that Metaverse is a new technology in healthcare and its entry into a hospital environment can affect all organizational aspects of the hospital*,* it is necessary to prepare for it before full establishment in the organization. Being ready to create or change ethical standards and security measures is very important. The issue of informed consent and confidentiality is very important.”*

Participant #13: *“Well*,* Metaverse is a new technology and a transformation in virtual interactions. I think the discussion of data integrity and maintaining data encryption is a vital issue for working with this technology in the healthcare field. Pieces of training and exercises must be considered for the learning of the members of the organization in these cases.”*

Participant #16: *“It is important for healthcare professionals to understand the implications of using metaverse technology in their practice*,* including the importance of maintaining patient privacy*,* upholding ethical standards*,* and adhering to professional guidelines.”*

Individual readiness and the ability to identify individual aspects of the metaverse were the most crucial and effective aspects of the code “internal and external considerations of digital professionalism” for the metaverse in healthcare, indicating that healthcare specialists need to be prepared for these considerations and able to identify the aspects faced.

Participant #6: *“Individual readiness is the first level of ability to develop digital professionalism. In my opinion*,* digital professionalism skills are acquired when one increases understanding and confidence in the use of digital technology”*.

Participant #14: *“I think the Ethical Use of Virtual Environments is very important. A professional in the field of healthcare should not behave in a Discriminatory or offensive way when using any new technology (metaverse).”*

Participant #17: *“Individual readiness refers to the preparedness of healthcare professionals to adapt to and engage with metaverse technology. It involves their technological proficiency*,* willingness to learn and embrace new tools*,* and their understanding of the potential benefits and challenges associated with the metaverse. A strong foundation in digital literacy is crucial for healthcare specialists to effectively utilize metaverse technology in their practice.”*

Participant #9: *“I believe that cultivating an attitude of fearlessness and responsible use of technology is one of the key characteristics of individuality. When it comes to using new technology*,* most healthcare professionals are a little cautious and a little worried. I think increasing curiosity can be very effective in improving an individual readiness to embrace technology like the Metaverse”*.

Participant #19: *“Healthcare specialists need to be able to identify individual aspects that may impact their use of the metaverse. This includes considerations such as their own comfort level with technology*,* their personal ethical standards*,* and their ability to maintain patient confidentiality and privacy in virtual environments. Being able to identify these individual aspects enables healthcare professionals to address any gaps in their knowledge or skills*,* seek appropriate training and support*,* and ensure ethical and professional conduct while using the metaverse.”*

The code “internal and external considerations of digital professionalism” in the metaverse in healthcare highlights the crucial aspect of social readiness and identifying social aspects. This indicates that healthcare specialists must possess social readiness and the ability to identify relevant aspects to effectively address these considerations.

Participant #11: *“I believe that society has a great impact on creating acceptance of this technology (Metaverse). So the social environment must be a favorable environment. I think the medical society should always be ready for any new technology”*.

Participant #12: *“We should comprehend that successful adaptation will be contingent on societal readiness just as much as on technical ones. Collaboration and Teamwork*,* Professional Conduct*,* and Communication all are cases of societal readiness.”*

Participant #15: *“The metaverse in healthcare offers promising opportunities for enhancing social aspects of healthcare delivery. By bridging distances*,* creating virtual support systems*,* and enabling immersive training experiences*,* the metaverse has the potential to transform healthcare by improving access*,* communication*,* and overall patient experience.”*

Participant #20: *“For the metaverse to be effectively integrated into healthcare*,* social readiness is crucial. There must be acceptance and readiness among both patients and healthcare professionals to utilize and trust this technology. Additionally*,* privacy and security concerns need to be addressed to ensure the confidentiality of patient data and protect against potential breaches.”*

### Early foundations

Preparing lessons for teaching was the most crucial and effective aspect of the code “early foundation of digital professionalism” for the metaverse in healthcare, indicating that healthcare specialists need to be prepared to teach basic digital technology.

Participant #1: *“I believe that we can move towards digital professionalism when that technology is taught at an advanced level. In fact teaching and preparing lessons on basic digital technology is indeed a crucial and effective aspect when establishing the early foundations of digital professionalism in healthcare*,* especially in relation to the metaverse.”*

Participant #4: *“I really feel the lack of basic digital technology training in medical departments. As the metaverse represents a relatively new and evolving technology*,* many healthcare professionals may not be familiar with its intricacies or how to effectively utilize it in their practice. Therefore*,* it becomes essential for healthcare specialists to be prepared and equipped with the knowledge and skills to teach their colleagues and other healthcare professionals about the basics of digital technology and the metaverse.”*

Participant #5: *“Digital technologies (e.g.*,* metaverse*,* artificial intelligence) are becoming mainstream and it is essential that nurses have the readiness to teach in the development or implementation of these emerging technologies. This shows the importance of training and paying more attention to digital professionalism. By teaching basic digital technology*,* healthcare specialists can empower their peers to embrace and adopt new technologies securely and effectively. This includes providing guidance on how to navigate virtual environments*,* understanding the connectivity and hardware requirements*,* and explaining the basic functionalities and operations within the metaverse.”*

In the code “early foundations of digital professionalism” for the metaverse in healthcare, professional development in learning is the most crucial and effective aspect, emphasizing the need for healthcar*e professionals to stay updated*.

Participant #7: *“A physician or nurse should not be afraid to use new technologies. I think practicing in a simulated environment encourages learning and using that technology. You know*,* I say that she or he (physician or nurse) should try to update and stay updated. Indeed*,* professional development in learning is a crucial and effective aspect when laying the early foundations of digital professionalism in the context of the metaverse in healthcare. It emphasizes the need for healthcare professionals to continually update their knowledge and skills to keep pace with new advancements and technologies”*.

Participant #8: *“Embrace any new technology requires the acceptance of professionals who know and are learned in continuous learning and being aware of potential aspects. Engaging in continuous learning allows healthcare professionals to understand the potential benefits and risks associated with the metaverse*,* enabling them to make informed decisions in their professional practice. They can gain a deeper understanding of the ethical considerations*,* privacy concerns*,* and legal obligations that come with using this technology.”*

Participant #10: *“Nothing beats being in an environment for learning*,* if we are looking for a digital professional who is able to cope with new technologies such as Metaverse*,* we must pay special attention to the professional development in learning. professional development in learning is a critical and effective aspect when establishing the early foundations of digital professionalism in the metaverse in healthcare. It supports healthcare professionals in staying updated*,* acquiring necessary skills*,* and fostering a mindset of lifelong learning. By doing so*,* healthcare professionals can optimize the use of metaverse technology to provide quality healthcare services in a rapidly evolving digital landscape.”*

## Discussion

This study has made a substantial contribution by employing semi-structured online interviews to identify and elucidate the core principles of digital professionalism, which are essential for implementing metaverse technology in healthcare. These principles have emerged from the valuable insights and perspectives provided by experts working in this field. The findings emphasize the importance of considering various aspects to foster digital professionalism when using the metaverse in healthcare settings.

Organizational preparedness plays a crucial role in ensuring that institutions are equipped with the necessary resources to adapt effectively to new technological advancements. Additionally, individual readiness is essential; individuals should be willing to embrace technology at any level of proficiency and exhibit curiosity and fearlessness towards learning novel digital tools. Social readiness is another critical aspect where healthcare specialists need awareness of societal implications for employing metaverse solutions.

Prior research has indicated that digital professionalism requires organizational and individual preparedness to navigate evolving technological landscapes effectively. At an individual level, readiness entails being open to embracing technology regardless of one’s technical proficiency. It also involves having a sense of curiosity and fearlessness when acquiring new digital skills. Moreover, individuals with expertise in their professional domain can contribute significantly by assisting, guiding, and educating others on developing digital professionalism [11–13].

In addition to individual readiness, environmental factors within a healthcare setting play a crucial role in facilitating users’ acceptance of novel technologies. The environment fostered by colleagues or management significantly shapes individuals’ attitudes towards these emerging technologies [12, 14]. Therefore, creating an environment that promotes openness and encourages exploration is essential for fostering adoption.

By focusing on both organizational and individual levels of preparedness, healthcare professionals can ensure the successful integration of metaverse technologies into their practice while upholding principles associated with digital professionalism.

In the realm of digital professionalism for metaverse in healthcare, it is imperative to consider the principles of social readiness. Awareness and addressing these principles can significantly contribute to upholding a commendable level of digital professionalism within this context. A fundamental aspect that plays a pivotal role in attaining such professionalism entails imparting knowledge of technology fundamentals through education. By pursuing this approach, individuals are empowered with the foundational understanding required to foster skill development and competence in navigating and utilizing various digital technologies [13, 15]. Healthcare professionals operating within the metaverse or virtual reality landscapes explicitly related to healthcare delivery, treatment interventions, or research endeavours must remain abreast of all developments regarding new technological advancements.

Based on the findings from our study, it is clear that careful planning and establishing a supportive learning environment are essential components in fostering digital professionalism within emerging technologies such as the metaverse. Previous research has highlighted a dearth of standardized principles and methodologies to enhance digital professionalism among nursing and medical students. Moreover, specific countries lack international, national, or local guidelines that outline proper conduct when engaging with digital technology [15–17]. Consequently, uniform approaches to cultivating practical and ethical behaviours are absent in this context.

Emerging evidence suggests that educators should prioritize meticulous planning processes to integrate digital professionalism principles comprehensively into educational curricula concerning emerging technologies like the metaverse. By doing so, educators and learners will be better equipped to navigate challenges associated with these transformative technological platforms.

The present study’s findings indicate that the principles of digital professionalism for the metaverse in healthcare can be organized into ten significant categories. These essential principles encompass a variety of areas such as privacy and security, ensuring informed consent from patients, promoting trustworthiness and ethical behaviour, championing accessibility and inclusivity, maintaining professional boundaries, relying on evidence-based practice to inform decision-making processes, continuously enhancing education and training efforts to stay up-to-date with emerging technologies within the metaverse context. Additionally, fostering collaboration by encouraging interoperability among different systems is crucial for exchanging vital information between healthcare professionals. Furthermore, feedback mechanisms are crucial in facilitating improvement initiatives while complying with regulatory standards to ensure appropriate conduct following prevailing legal guidelines.

Ensuring patient privacy and safeguarding data security are paramount in healthcare practices. In order to uphold the confidentiality of sensitive patient information [18, 19], robust security measures such as encryption protocols and access controls must be implemented. Furthermore, obtaining informed consent from patients should be considered a fundamental principle when utilizing patients’ health records in Metaverse environments [20–22].

Before collecting or using individuals’ health data within virtual healthcare settings like metaverse platforms, explicit informed consent should be obtained from each patient. This process allows for comprehensive explanations about the purpose of data usage within these virtual realms, highlighting potential benefits that may arise while addressing all associated aspects of their participation. By adhering to this crucial principle of digital professionalism, healthcare professionals can ensure ethical practice and protect patient privacy in the rapidly evolving landscape of technology-driven care.

Trust, honesty, and integrity are essential in ensuring patient confidentiality within the metaverse. Healthcare professionals are responsible for establishing a solid foundation of trust with patients regarding the sensitive nature of their personal health information [11, 12]. Moreover, accessibility and inclusion play fundamental roles in virtual healthcare settings by providing equal opportunities for individuals from diverse backgrounds [23, 24] or with differing abilities. In this context, accessibility refers to making virtual health services readily available to many individuals, including those with physical, sensory or cognitive disabilities. By prioritizing accessibility and inclusion within digital platforms used for healthcare provision in the metaverse, healthcare providers can extend their reach while promoting equitable experiences throughout each individual’s healthcare journey.

Professional boundaries in a professional environment are crucial for establishing acceptable and ethical behaviour [25, 26]. In the metaverse, it is imperative to maintain appropriate professional boundaries between healthcare providers and patients. Providing virtual healthcare services within this digital space requires adherence to evidence-based practices. It is essential to avoid any form of promotion or endorsement of unproven or pseudoscientific treatments or therapies. Instead, virtual healthcare services should integrate the most reliable evidence from research studies along with clinical expertise while considering individual patient values and preferences. This approach enables effective decision-making regarding treatment options and facilitates the delivery of high-quality care.

Maintaining professional boundaries helps ensure that interactions between healthcare providers and patients adhere to established ethical standards. Healthcare professionals must prioritize trust, integrity, confidentiality, informed consent, as well as patient privacy when utilizing technology like the metaverse for delivering medical care virtually.

Informed consent is integral in upholding patient autonomy and safeguarding their rights in using personal health information within metaverse settings [11]. Obtaining explicit informed consent from individuals becomes paramount before utilizing patients’ data for storage or analysis purposes on virtual platforms such as the metaverse [11, 27].

Patient privacy is an utmost priority when embracing technologies like the metaverse for virtual healthcare services. A multifaceted approach to digital professionalism in the metaverse also includes continuous education and training for healthcare professionals [28].

Continuous education and training, also referred to as continuing education or professional development, pertains to the continual acquisition of knowledge, skills, and competencies by individuals within their specific fields [29]. Healthcare professionals engaged in the metaverse setting must receive ongoing training and education to remain well-informed about emerging technologies and advancements in digital health practices. Continuous education and training initiatives aim to augment professional competence and enhance job performance quality while remaining up-to-date with the latest developments pertinent to a particular discipline [30].

Another essential principle to consider is the significance of cultivating collaboration and guaranteeing interoperability. Collaboration involves individuals or groups working together towards a common goal [31]. Conversely, interoperability refers to the ability of different systems, technologies, or organizations to interact smoothly and exchange information [32]. Encouraging collaboration and interoperability among diverse healthcare systems and platforms within the metaverse is vital. This will facilitate the seamless flow of patient data exchange and ensure uninterrupted care provision.

Feedback and improvement are interconnected concepts crucial in personal and professional growth, skill development, and continuous learning [33, 34]. To improve virtual healthcare experiences in the metaverse, actively listen to concerns, address any issues promptly, and adapt practices based on these insights.

To ensure adherence to laws, regulations, guidelines, and standards set by regulatory bodies or authorities within a specific industry or jurisdiction [35, 36], compliance with the relevant legal requirements and standards governing virtual healthcare services in the metaverse is imperative.

### Limitations

There are a few limitations to note for this study. Due to certain limitations in our study design, we could not include online interviews with other healthcare professionals, such as physicians and nurses. This limitation restricted our insights into their perspectives on access issues. Consequently, there may be some inherent bias towards digital technologies since we only interviewed medical information and health informatics specialists with expertise in this domain. To mitigate this potential bias in future research projects investigating similar areas of interest, we think it would be advisable to expand the scope of interviews by including a broader range of specialists. It may also be beneficial to employ thematic analysis techniques for an enriched understanding while comparing interview texts.

While the authors successfully identified ten essential principles of digital professionalism for the metaverse in healthcare, including Privacy and Security, Informed Consent, Trust and Integrity, Accessibility and Inclusion, Professional Boundaries, Evidence-Based Practice, Continuous Education and Training, Collaboration and Interoperability, Feedback and Improvement, and Regulatory Compliance, the study lacks a cross-analysis of these principles based on the varying levels of development in the countries where the interviewed individuals reside. The absence of this analysis may limit the overall comprehensiveness of the study and its applicability across diverse healthcare contexts, but also offers opportunity for future study.

## Conclusion

As the metaverse continues to evolve and permeate various industries, including healthcare, establishing principles of digital professionalism becomes crucial to ensure ethical and responsible practices. This study was conducted to explore some key principles of this new landscape. The results of this study indicate that protecting patient privacy and maintaining data security within the metaverse should be the top priority (i.e., Privacy and Security). Clearly explain the purpose, potential benefits, and aspects associated with using patients’ data in virtual healthcare settings (i.e., Informed Consent). Provide accurate and reliable information, ensuring that healthcare professionals and avatars in the metaverse adhere to ethical practices (i.e., Trust and Integrity). Providing virtual healthcare services and resources are available to individuals of diverse backgrounds, ethnicities, ages, genders, and socioeconomic statuses (i.e., Accessibility and Inclusion). Avoid engaging in any personal relationships or inappropriate interactions that could compromise professional judgment (i.e., Professional Boundaries). Virtual healthcare services offered within the metaverse should be evidence-based and supported by scientific research (i.e., Evidence-Based Practice). Healthcare professionals involved in the metaverse should receive ongoing training and education in order to stay updated with emerging technologies, digital health advancements, and best practices (i.e., Continuous Education and Training). Encourage collaboration and interoperability between different healthcare systems and platforms within the metaverse (i.e., Collaboration and Interoperability). Actively listen to concerns, address any issues promptly, and adapt practices based on these insights (i.e., Feedback and Improvement). Stay updated on changes in regulations to ensure adherence and mitigate legal risks (i.e., Regulatory Compliance). By adhering to these principles of digital professionalism, healthcare professionals can ensure ethical practice, protect patient privacy, and provide effective care within the metaverse.

### Electronic supplementary material

Below is the link to the electronic supplementary material.

Supplementary Material 1

Supplementary Material 2

## Data Availability

Please contact the corresponding author if you would like access to the datasets used and/or analyzed during this study.
