# Supplementary material for: Principles of digital professionalism for the metaverse in healthcare
Source: BMC Med Inform Decis Mak. 2024 Jul 22;24:201. doi: 10.1186/s12911-024-02607-y (PMC11265428; doi:10.1186/s12911-024-02607-y)
Supplement: Supplementary file 1 — Supplementary Material 1 [file 12911_2024_2607_MOESM1_ESM.docx]

## **Supplementary file 1**

**Conducted interviews**

## **Internal and external considerations**

**Interviewer**: Today we have with us an expert in the field of digital technology and healthcare. Thank you for joining us. Can you please share your thoughts on the adoption and utilization of digital technology by healthcare providers?

**Participant #2**: Thank you for having me. The adoption and utilization of digital technology by healthcare providers, such as physicians and nurses, is an important aspect of modern healthcare. While there have been significant advancements in technology in recent years, the healthcare industry has been slower to embrace these changes compared to other professions.

**Interviewer**: What are some of the reasons for this slower adoption?

**Participant #2**: There are several reasons for the slower adoption of digital technology in healthcare. One of the main reasons is the resistance to change. Healthcare professionals are often busy with their daily responsibilities and may be hesitant to incorporate new technologies into their practice. This resistance can stem from a lack of familiarity with these technologies or concerns about their impact on workflow and patient care.

**Interviewer**: How can healthcare organizations address this resistance and promote the adoption of digital technology?

**Participant #2**: To address this resistance, healthcare organizations need to prioritize organizational readiness. This involves providing comprehensive training and resources to healthcare professionals to ensure they have the necessary skills to utilize digital technologies effectively. It also includes implementing policies and protocols that address concerns related to security and privacy of patient information.

**Interviewer**: Can you elaborate on the importance of organizational readiness in adopting digital technology?

**Participant #2**: Organizational readiness is crucial for successful adoption and utilization of digital technology. It involves creating an environment that supports and encourages the use of these technologies. This can be achieved by providing the necessary infrastructure and resources, fostering a culture of innovation and continuous learning, and ensuring interoperability between different systems. It also includes addressing concerns about data privacy and security through robust security measures and ongoing training for healthcare professionals.

**Interviewer**: What are some of the challenges that healthcare professionals face in adopting digital technology?

**Participant #2**: Healthcare professionals face several challenges in adopting digital technology. One challenge is the resistance to change and the fear of the unknown. Healthcare professionals may be hesitant to embrace new technologies due to concerns about their impact on their workflow and patient care. Additionally, the potential for increased workload and information overload can also be a challenge. Healthcare professionals need support in managing the influx of data and information that comes with digital technologies.

**Interviewer**: How can healthcare organizations support healthcare professionals in adopting digital technology?

**Participant #2**: Healthcare organizations can support healthcare professionals by providing comprehensive training programs that equip them with the necessary skills to utilize digital technologies effectively. They can also invest in systems and tools that help healthcare professionals navigate and process the influx of information. Additionally, organizations should create a culture that values innovation and continuous learning, where healthcare professionals feel supported and encouraged to embrace new technologies.

**Interviewer**: Thank you for sharing your insights on this topic. It is evident that organizational readiness plays a crucial role in the adoption and utilization of digital technology by healthcare providers. Healthcare organizations need to prioritize training, address concerns related to security and privacy, and create a culture that supports innovation and continuous learning.

**Interviewer**: In the context of digital professionalism for the metaverse in healthcare, can you elaborate on the importance of organizational readiness and identifying organizational risks?

**Participant #3**: Certainly. Organizational readiness is crucial when it comes to digital professionalism in the metaverse in healthcare. The metaverse, which refers to a virtual reality space where people can interact with each other and digital objects, is becoming increasingly relevant in healthcare. It offers opportunities for virtual consultations, medical training simulations, and patient education, among other applications.

To ensure digital professionalism in the metaverse, healthcare organizations need to be well-prepared and ready to embrace this technology. This involves having the necessary infrastructure, resources, and policies in place to support the use of the metaverse in healthcare settings. It also includes providing training and education for healthcare specialists to navigate and utilize the metaverse effectively.

Identifying organizational risks is another important aspect of digital professionalism in the metaverse. Healthcare organizations need to proactively assess and address potential risks associated with using the metaverse in healthcare. This includes considering factors such as data security and privacy, ethical considerations, and potential challenges related to virtual interactions. By identifying and addressing these risks, organizations can ensure the safe and responsible use of the metaverse in healthcare.

**Interviewer**: How can healthcare specialists be well-prepared and capable of recognizing and addressing these considerations proactively?

**Participant #3**: Healthcare specialists can be well-prepared by staying informed and educated about the metaverse and its potential applications in healthcare. This involves keeping up with the latest developments in virtual reality and understanding how it can be integrated into their practice. Healthcare specialists should also seek out training opportunities and resources that can help them develop the necessary skills to navigate and utilize the metaverse effectively.

Recognizing and addressing considerations proactively requires a proactive mindset and a willingness to stay informed about potential risks and challenges. Healthcare specialists should actively engage in discussions and forums related to digital professionalism in the metaverse. They should also collaborate with their colleagues and healthcare organizations to identify and address any organizational risks associated with using the metaverse in healthcare.

By being well-prepared and proactive, healthcare specialists can ensure that they are equipped to navigate the metaverse responsibly and professionally, while providing high-quality care to their patients.

**Interviewer**: Thank you for highlighting the importance of organizational readiness and identifying organizational risks in the context of digital professionalism for the metaverse in healthcare. It is clear that being well-prepared and proactive is essential for healthcare specialists to embrace this technology and address any associated considerations effectively.

**Interviewer**: We have our expert back with us to discuss the importance of data integrity and maintaining data encryption in the context of the metaverse in healthcare. Thank you for joining us again. Can you elaborate on the significance of these aspects?

**Participant #13**: Thank you for having me again. You are absolutely right, data integrity and maintaining data encryption are crucial considerations when working with the metaverse in the healthcare field. The metaverse, being a virtual environment, involves the exchange and storage of sensitive healthcare data, making it essential to ensure the security and integrity of that data.

Data integrity refers to the accuracy, consistency, and reliability of the information stored and transmitted within the metaverse. It is vital to ensure that healthcare data remains intact and unaltered throughout its lifecycle. This includes protecting against unauthorized modifications, ensuring data quality, and maintaining the trustworthiness of the information.

Maintaining data encryption is equally important in safeguarding healthcare data in the metaverse. Encryption involves encoding the data in a way that renders it unreadable to unauthorized individuals. By implementing strong encryption protocols, healthcare professionals can protect sensitive patient information from being accessed or intercepted by unauthorized parties.

Training and exercises play a crucial role in ensuring that healthcare professionals are well-prepared to handle data integrity and encryption in the metaverse. Training programs can educate healthcare professionals about the importance of data security, best practices for encryption, and guidelines for maintaining data integrity. Exercises and simulations can provide hands-on experience in handling and securing sensitive data in virtual environments.

By prioritizing data integrity and maintaining data encryption, healthcare organizations can ensure the privacy, confidentiality, and security of patient information in the metaverse.

**Interviewer**: How can healthcare organizations effectively train their members to address data integrity and maintain data encryption in the metaverse?

**Participant #13**: Healthcare organizations can adopt various strategies to effectively train their members in addressing data integrity and maintaining data encryption in the metaverse. First and foremost, organizations should provide comprehensive training programs that cover the fundamentals of data security, encryption techniques, and protocols specific to the metaverse.

These training programs can be delivered through various methods, such as online modules, workshops, or seminars. It is important to ensure that the training materials are up-to-date and reflect the latest advancements in data security and encryption technologies.

Additionally, healthcare organizations should conduct regular exercises and simulations to test the knowledge and skills of their members in handling data integrity and encryption in the metaverse. These exercises can involve scenarios that mimic real-world situations, allowing healthcare professionals to practice their response and decision-making skills in a safe and controlled environment.

Furthermore, healthcare organizations should establish clear policies and guidelines regarding data integrity and encryption in the metaverse. These policies should outline the expectations, responsibilities, and procedures to be followed by all members of the organization. Regular communication and reminders about these policies can help reinforce the importance of data security and encryption.

**Interviewer**: Thank you for highlighting the significance of data integrity and maintaining data encryption in the metaverse for healthcare organizations. It is evident that training and exercises are essential in ensuring that healthcare professionals are equipped to handle data security effectively and maintain the privacy and integrity of patient information in virtual environments.

**Interviewer**: We have our expert back with us to discuss the implications of using metaverse technology in healthcare practice, particularly regarding patient privacy, ethical standards, and professional guidelines. Thank you for joining us again. Can you elaborate on the importance of these aspects?

**Participant #16**: Thank you for having me again. You are absolutely right, healthcare professionals must understand the implications of using metaverse technology in their practice, especially in relation to patient privacy, ethical standards, and professional guidelines. The adoption of metaverse technology brings new opportunities and challenges that need to be addressed responsibly.

First and foremost, patient privacy is of utmost importance when using metaverse technology in healthcare. Healthcare professionals must ensure that patient information is securely stored and transmitted within the virtual environment. This includes implementing robust security measures, such as data encryption and access controls, to protect patient data from unauthorized access or breaches.

Ethical standards play a crucial role in guiding healthcare professionals' behavior and decision-making in the metaverse. They must uphold the principles of beneficence, non-maleficence, autonomy, and justice when using technology. This includes obtaining informed consent from patients for their participation in virtual interactions, maintaining confidentiality, and respecting patients' autonomy and privacy preferences.

Adhering to professional guidelines is essential to ensure that healthcare professionals use metaverse technology in a responsible and ethical manner. Professional organizations and regulatory bodies often provide guidelines and standards specific to the use of technology in healthcare. Healthcare professionals should familiarize themselves with these guidelines and ensure that their practices align with the recommended best practices.

By prioritizing patient privacy, upholding ethical standards, and adhering to professional guidelines, healthcare professionals can maintain trust with their patients and promote the responsible use of metaverse technology in healthcare.

**Interviewer**: How can healthcare professionals ensure they are maintaining patient privacy, upholding ethical standards, and adhering to professional guidelines when utilizing metaverse technology?

**Participant #16**: Healthcare professionals can take several steps to ensure they are maintaining patient privacy, upholding ethical standards, and adhering to professional guidelines when utilizing metaverse technology.

Firstly, they should undergo training and education on the ethical implications and privacy considerations associated with using metaverse technology in healthcare. This will enable them to understand the potential risks and develop strategies to mitigate them effectively.

Secondly, healthcare professionals should familiarize themselves with the relevant legal and regulatory requirements pertaining to patient privacy in the metaverse. They should ensure that the technology they use complies with these requirements and that appropriate safeguards are in place to protect patient data.

Additionally, healthcare professionals should obtain informed consent from patients before engaging in virtual interactions. This includes explaining the purpose, benefits, and potential risks associated with the use of metaverse technology and obtaining patients' consent to participate.

Regular self-reflection and evaluation of their practices can help healthcare professionals identify any ethical dilemmas or privacy concerns that may arise when using metaverse technology. They should seek guidance from ethics committees or consult with colleagues when faced with challenging situations.

Lastly, staying updated with professional guidelines and best practices is crucial. Healthcare professionals should regularly review and incorporate the latest recommendations into their practice, ensuring they are providing care in accordance with the highest ethical and professional standards.

**Interviewer**: Thank you for emphasizing the importance of patient privacy, ethical standards, and professional guidelines when utilizing metaverse technology in healthcare practice. It is evident that healthcare professionals must prioritize these aspects to ensure responsible and ethical use of technology while maintaining patient trust and confidentiality.

**Interviewer**: We have our expert back with us to discuss the first level of ability in developing digital professionalism, which is individual readiness. Thank you for joining us again. Can you elaborate on the importance of individual readiness in acquiring digital professionalism skills?

**Participant #6**: Thank you for having me again. Individual readiness is indeed a crucial aspect when it comes to developing digital professionalism skills. It refers to an individual's level of preparedness, understanding, and confidence in using digital technology effectively and responsibly.

Increasing understanding and confidence in the use of digital technology is essential for healthcare professionals to develop digital professionalism skills. It involves acquiring the necessary knowledge and skills to navigate and utilize digital tools and platforms in their practice. This includes understanding the functionalities of various digital technologies, such as electronic health records, telemedicine platforms, and communication tools.

By increasing understanding, healthcare professionals can effectively leverage digital technology to enhance patient care, streamline workflows, and improve communication and collaboration with colleagues. It allows them to harness the full potential of digital tools and platforms, leading to more efficient and effective healthcare delivery.

Confidence in using digital technology is equally important. When healthcare professionals feel confident in their ability to navigate and utilize digital tools, they are more likely to embrace and integrate them into their practice. Confidence enables healthcare professionals to overcome any apprehension or resistance to change, allowing them to fully embrace the opportunities that digital technology offers.

Acquiring digital professionalism skills through individual readiness is an ongoing process. It requires a commitment to continuous learning, staying updated with emerging technologies, and seeking opportunities to expand one's digital skill set. By investing in individual readiness, healthcare professionals can adapt to the evolving digital landscape and provide the best possible care in the digital era.

**Interviewer**: How can healthcare professionals enhance their individual readiness and acquire digital professionalism skills?

**Participant #6**: Healthcare professionals can enhance their individual readiness and acquire digital professionalism skills through various approaches. Here are a few suggestions:

1. Continuous learning: Actively seek out educational opportunities, such as workshops, webinars, and online courses, that focus on digital technology in healthcare. These learning opportunities can provide insights, best practices, and hands-on experiences to enhance digital proficiency.

2. Self-directed learning: Take advantage of online resources, tutorials, and documentation provided by technology vendors and reputable sources. These resources can help healthcare professionals explore and master specific digital tools and platforms.

3. Peer collaboration: Engage in discussions and collaborations with colleagues who have expertise in digital technology. Sharing experiences, knowledge, and challenges can foster a supportive learning environment and promote the exchange of best practices.

4. Experimentation and practice: Take the initiative to explore and experiment with different digital tools and platforms. Practice using them in a safe environment to build confidence and familiarity.

5. Seek mentorship: Find mentors or digital champions within the healthcare field who can provide guidance, support, and mentorship in developing digital professionalism skills.

6. Stay updated: Regularly stay informed about the latest advancements and trends in digital technology in healthcare. Subscribe to relevant newsletters, join professional communities, and follow reputable sources to stay updated with emerging technologies and best practices.

By actively engaging in these strategies, healthcare professionals can enhance their individual readiness, increase their understanding and confidence in using digital technology, and ultimately acquire digital professionalism skills.

**Interviewer**: Thank you for emphasizing the importance of individual readiness in acquiring digital professionalism skills. It is evident that healthcare professionals must actively invest in their own learning and development to effectively navigate and utilize digital technology in their practice.

**Interviewer**: We have our expert back with us to discuss the importance of ethical use of virtual environments, particularly in the context of healthcare professionals using new technologies like the metaverse. Thank you for joining us again. Can you elaborate on the significance of ethical behavior and the avoidance of discriminatory or offensive behavior in utilizing new technologies?

**Participant #14**: Thank you for having me again. You're absolutely right, ethical behavior and the avoidance of discriminatory or offensive behavior are crucial when healthcare professionals use new technologies, including the metaverse. It is essential for healthcare professionals to uphold ethical standards and treat all individuals with respect and fairness, regardless of the virtual environment they are in.

In virtual environments, healthcare professionals interact with patients, colleagues, and other participants. It is important to remember that these interactions should mirror the same professionalism and ethical conduct as in face-to-face interactions. Discriminatory or offensive behavior is unacceptable and can undermine the trust, dignity, and well-being of individuals involved.

Healthcare professionals should adhere to ethical principles, such as respect for autonomy, non-maleficence, beneficence, and justice, when using new technologies. This includes treating all individuals with dignity and respect, maintaining confidentiality and privacy, and ensuring equal access to healthcare services.

Moreover, healthcare professionals must be aware of potential biases and prejudices that may influence their behavior in virtual environments. It is crucial to recognize and challenge these biases to ensure fair and equitable treatment for all individuals, regardless of their background, identity, or characteristics.

By promoting ethical behavior and avoiding discriminatory or offensive conduct, healthcare professionals can create a safe and inclusive virtual environment that fosters trust, respect, and patient-centered care.

**Interviewer**: How can healthcare professionals ensure ethical behavior and avoid discriminatory or offensive behavior when utilizing new technologies like the metaverse?

**Participant #14**: Healthcare professionals can take several steps to ensure ethical behavior and avoid discriminatory or offensive conduct when utilizing new technologies like the metaverse:

1. Education and awareness: Healthcare professionals should undergo training and education on ethical guidelines and standards specific to the use of technology in healthcare. This includes understanding the potential biases, challenges, and ethical considerations that may arise in virtual environments.

2. Reflective practice: Engage in self-reflection and introspection to identify potential biases, prejudices, or assumptions that may affect interactions in virtual environments. Regularly evaluate and challenge these biases to ensure fair and equitable treatment for all individuals.

3. Cultural competence: Develop cultural competence and sensitivity to better understand and respect diverse perspectives, values, and beliefs. This includes being aware of cultural differences, practicing active listening, and adapting communication styles to foster effective and inclusive virtual interactions.

4. Professional guidelines: Familiarize themselves with professional guidelines and codes of ethics that address the use of technology in healthcare. Adhere to these guidelines in all virtual interactions and ensure that ethical principles are upheld.

5. Reporting mechanisms: Healthcare professionals should be aware of reporting mechanisms within their organization to address any instances of discriminatory or offensive behavior. They should report any concerns promptly and participate in creating a safe and inclusive virtual environment.

6. Peer support and collaboration: Engage in discussions and collaborations with colleagues to share experiences, challenges, and best practices related to ethical behavior in virtual environments. Support and learn from each other to create a culture of ethical conduct.

By actively implementing these strategies, healthcare professionals can ensure ethical behavior, avoid discriminatory or offensive conduct, and create a virtual environment that upholds the values of respect, inclusivity, and patient-centered care.

**Interviewer**: Thank you for emphasizing the importance of ethical behavior and the avoidance of discriminatory or offensive conduct in utilizing new technologies like the metaverse in healthcare. It is evident that healthcare professionals must prioritize ethical guidelines and foster an inclusive virtual environment to provide equitable and patient-centered care.

**Interviewer**: We have our expert back with us to discuss individual readiness in relation to metaverse technology in healthcare. Thank you for joining us again. Can you elaborate on the importance of individual readiness and digital literacy in effectively utilizing metaverse technology?

**Participant #17**: Thank you for having me again. You're absolutely right, individual readiness and digital literacy are key factors in effectively utilizing metaverse technology in healthcare practice. Individual readiness refers to the preparedness and willingness of healthcare professionals to adapt to and engage with new technologies like the metaverse.

Technological proficiency is a crucial component of individual readiness. Healthcare professionals need to have a solid foundation in digital literacy, which includes having the necessary skills to navigate and utilize digital tools and platforms effectively. This includes understanding basic computer operations, using software applications, and being familiar with internet-based technologies.

Digital literacy also involves being able to critically evaluate and use information obtained from digital sources. Healthcare professionals must be able to discern reliable and trustworthy sources of information within the metaverse to make informed decisions and provide evidence-based care.

Willingness to learn and embrace new tools is another important aspect of individual readiness. Healthcare professionals need to have an open mindset and a proactive approach to learning and adopting new technologies. This includes being receptive to training opportunities, exploring different features and functionalities, and actively seeking ways to enhance their digital skills.

Understanding the potential benefits and challenges associated with the metaverse is also essential for individual readiness. Healthcare professionals should be aware of how metaverse technology can improve patient care, enhance communication and collaboration, and streamline workflows. At the same time, they should also understand the potential challenges, such as privacy and security concerns, and be prepared to address them appropriately.

By developing individual readiness and digital literacy, healthcare professionals can effectively utilize metaverse technology in their practice, leading to improved patient outcomes, enhanced efficiency, and better overall healthcare delivery.

**Interviewer**: How can healthcare professionals enhance their individual readiness and develop digital literacy in relation to metaverse technology?

**Participant #17**: Healthcare professionals can take several steps to enhance their individual readiness and develop digital literacy in relation to metaverse technology:

1. Continuous learning: Actively seek out educational opportunities, such as workshops, webinars, and online courses, that focus on digital literacy and metaverse technology in healthcare. These learning opportunities can provide insights, hands-on experience, and practical guidance to enhance digital proficiency.

2. Technology exploration: Take the initiative to explore and familiarize oneself with different metaverse platforms and tools. Experiment with their features and functionalities to gain practical experience and build confidence in utilizing them.

3. Collaborative learning: Engage in discussions and collaborations with colleagues who have experience with metaverse technology. Share knowledge, exchange best practices, and learn from each other's experiences to enhance digital literacy collectively.

4. Professional networks and communities: Join professional networks and communities focused on digital health or metaverse technology. These platforms provide opportunities to connect with experts, share resources, and participate in discussions and events related to digital literacy and metaverse technology.

5. Stay updated: Regularly stay informed about the latest advancements and trends in metaverse technology in healthcare. Subscribe to relevant newsletters, follow thought leaders, and participate in conferences or webinars to stay updated with emerging technologies and best practices.

6. Seek mentorship: Find mentors or digital champions within the healthcare field who can provide guidance, support, and mentorship in developing digital literacy and utilizing metaverse technology effectively.

By actively engaging in these strategies, healthcare professionals can enhance their individual readiness, develop digital literacy, and effectively utilize metaverse technology to improve patient care and healthcare outcomes.

**Interviewer**: Thank you for highlighting the importance of individual readiness and digital literacy in effectively utilizing metaverse technology in healthcare. It is evident that healthcare professionals must actively invest in their own learning and development to navigate and utilize digital tools effectively in their practice.

**Interviewer**: We have our expert back with us to discuss the importance of cultivating fearlessness and curiosity in individual readiness to embrace new technologies like the metaverse in healthcare. Thank you for joining us again. Can you elaborate on the significance of fearlessness and curiosity in improving individual readiness?

**Participant #9**: Thank you for having me again. You're absolutely right, cultivating fearlessness and curiosity is vital in improving individual readiness to embrace new technologies like the metaverse in healthcare. These characteristics play a crucial role in overcoming hesitations and fostering a proactive approach towards technology adoption.

Fearlessness involves being willing to step outside one's comfort zone and embrace new technologies with an open mind. Many healthcare professionals may feel cautious or worried when it comes to using new technology, as it involves a learning curve and potential challenges. However, cultivating fearlessness allows individuals to approach these technologies with confidence and a willingness to explore their potential benefits.

By cultivating fearlessness, healthcare professionals can overcome any apprehensions or resistance to change. This mindset enables them to adapt to new technologies more readily, actively engage in learning opportunities, and explore the possibilities that technologies like the metaverse offer for improving patient care and healthcare delivery.

Curiosity is another important characteristic that drives individual readiness. A curious mindset encourages healthcare professionals to ask questions, seek answers, and explore the capabilities and potential of new technologies. It fuels a desire to learn and understand how these technologies can be effectively utilized in their practice.

Increasing curiosity helps healthcare professionals to actively seek out information, resources, and training opportunities related to metaverse technology. It drives them to explore different use cases, understand the benefits and challenges, and envision how these technologies can enhance their own practice and improve patient outcomes.

By cultivating fearlessness and curiosity, healthcare professionals can enhance their individual readiness and embrace new technologies like the metaverse more effectively. These characteristics encourage a proactive approach to learning, experimentation, and collaboration, enabling healthcare professionals to harness the full potential of these technologies for the benefit of their patients and their own professional growth.

**Interviewer**: How can healthcare professionals cultivate fearlessness and curiosity in embracing new technologies like the metaverse?

**Participant #9**: Healthcare professionals can take several steps to cultivate fearlessness and curiosity in embracing new technologies like the metaverse:

1. Adopt a growth mindset: Embrace the belief that abilities and skills can be developed through dedication and hard work. This mindset encourages healthcare professionals to view challenges as opportunities for growth and learning, fostering fearlessness in the face of new technologies.

2. Seek learning opportunities: Actively seek out learning opportunities, such as workshops, webinars, and training programs, that focus on new technologies in healthcare. Engage in self-directed learning by exploring online resources, tutorials, and documentation related to the metaverse.

3. Embrace experimentation: Be open to experimenting with new technologies, including the metaverse. Engage in hands-on experiences, explore different features and functionalities, and learn from trial and error. Embracing experimentation fosters fearlessness and curiosity in understanding the potential of these technologies.

4. Connect with tech-savvy peers: Collaborate and learn from colleagues who are already familiar with the metaverse or other new technologies. Engage in discussions, share experiences, and seek their guidance and insights. This interaction can inspire curiosity and provide valuable learning opportunities.

5. Stay informed: Regularly stay updated with the latest trends, advancements, and success stories related to the metaverse and other new technologies in healthcare. Follow reputable sources, join professional communities, and participate in conferences or webinars to stay informed and foster curiosity.

6. Reflect and share experiences: Reflect on personal experiences and share insights with colleagues. Engage in discussions and knowledge-sharing activities to promote curiosity and continuous learning within the healthcare community.

By actively engaging in these strategies, healthcare professionals can cultivate fearlessness and curiosity, improving their individual readiness to embrace new technologies like the metaverse and drive positive change in healthcare practice.

**Interviewer**: Thank you for highlighting the importance of cultivating fearlessness and curiosity in improving individual readiness to embrace new technologies like the metaverse. It is evident that these characteristics play a significant role in overcoming hesitations, fostering a proactive mindset, and effectively utilizing new technologies in healthcare practice.

**Interviewer**: We have our expert back with us to discuss the importance of healthcare specialists identifying individual aspects that may impact their use of the metaverse. Thank you for joining us again. Can you elaborate on the significance of identifying these individual aspects and how it enables healthcare professionals to ensure ethical and professional conduct while using the metaverse?

**Participant #19**: Thank you for having me again. You're absolutely right, healthcare specialists need to identify individual aspects that may impact their use of the metaverse. This includes factors such as their comfort level with technology, personal ethical standards, and ability to maintain patient confidentiality and privacy in virtual environments.

Identifying these individual aspects is crucial because it allows healthcare professionals to assess their own readiness and capabilities in utilizing the metaverse. It helps them recognize any gaps in their knowledge or skills that may hinder their effective and ethical use of this technology.

Firstly, understanding one's comfort level with technology is essential. Some healthcare professionals may be more technologically inclined and feel at ease with using digital tools, while others may be less comfortable or have limited experience. By recognizing their comfort level, healthcare professionals can address any gaps by seeking appropriate training and support to enhance their technological proficiency.

Secondly, personal ethical standards play a significant role in the use of the metaverse. Healthcare professionals must align their use of technology with their ethical obligations to ensure patient-centered care, privacy, and confidentiality. By identifying their ethical standards, healthcare professionals can make informed decisions about their use of the metaverse and ensure that their actions align with professional guidelines and codes of ethics.

Lastly, maintaining patient confidentiality and privacy is of utmost importance in virtual environments. Healthcare professionals need to be aware of the potential risks and challenges in safeguarding patient information while using the metaverse. By recognizing their ability to maintain confidentiality and privacy, healthcare professionals can take necessary precautions, such as using secure platforms, implementing appropriate access controls, and following data protection guidelines.

Identifying these individual aspects enables healthcare professionals to take proactive measures to address any gaps or concerns. They can seek appropriate training and support to enhance their technological proficiency, engage in ethical discussions and reflection to ensure their actions align with professional standards, and implement strategies to maintain patient confidentiality and privacy in virtual environments.

By addressing these individual aspects, healthcare professionals can ensure ethical and professional conduct while using the metaverse, providing high-quality care to their patients and upholding the trust and confidence placed in them.

**Interviewer**: Thank you for emphasizing the importance of healthcare professionals identifying individual aspects that may impact their use of the metaverse. It is evident that recognizing factors such as comfort with technology, personal ethical standards, and ability to maintain patient confidentiality and privacy allows healthcare professionals to address any gaps, seek appropriate support, and ensure ethical and professional conduct in utilizing the metaverse.

**Interviewer**: We have our expert back with us to discuss the impact of society on creating acceptance of technologies like the metaverse and the importance of the medical society being ready for new technologies. Thank you for joining us again. Can you elaborate on the significance of a favorable social environment and the readiness of the medical society in accepting new technologies?

**Participant #11**: Thank you for having me again. You're absolutely right, a favorable social environment and the readiness of the medical society are crucial in creating acceptance and successful adoption of new technologies like the metaverse.

A favorable social environment is important because it influences the attitudes, beliefs, and perceptions of individuals and communities towards new technologies. When society embraces and supports technological advancements, it fosters a positive environment that encourages adoption and utilization. This acceptance can be influenced by factors such as the perceived benefits of the technology, trust in the technology providers, and understanding of its potential impact on various aspects of life, including healthcare.

In the context of the metaverse, a favorable social environment would involve society recognizing the potential benefits of using this technology in healthcare, such as improved access to care, enhanced communication and collaboration, and innovative treatment options. It would also involve addressing any concerns or challenges associated with the metaverse, such as privacy and security, and ensuring that appropriate safeguards are in place.

The readiness of the medical society is equally important. Healthcare professionals, organizations, and institutions need to be prepared and open to embracing new technologies. This readiness involves having a culture of continuous learning and innovation, being open to change, and actively seeking opportunities to explore and adopt new technologies.

The medical society should prioritize staying updated with emerging technologies, engaging in discussions and collaborations, and actively participating in the development and implementation of new technologies in healthcare. This readiness ensures that healthcare professionals are equipped with the knowledge, skills, and support needed to effectively utilize technologies like the metaverse in their practice.

By creating a favorable social environment and fostering readiness within the medical society, healthcare professionals can benefit from the collective support, resources, and expertise of their peers. This collaborative approach enables the successful integration of new technologies, like the metaverse, into healthcare practice, leading to improved patient care, enhanced efficiency, and better overall healthcare delivery.

**Interviewer**: Thank you for highlighting the significance of a favorable social environment and the readiness of the medical society in accepting new technologies. It is evident that societal acceptance and the preparedness of healthcare professionals and organizations play a crucial role in successful adoption and utilization of technologies like the metaverse in healthcare.

**Interviewer**: We have our expert back with us to discuss the importance of societal readiness in the successful adaptation of new technologies. Thank you for joining us again. Can you elaborate on the significance of collaboration and teamwork, professional conduct, and communication as examples of societal readiness?

**Participant #12**: Thank you for having me again. You're absolutely right, societal readiness is essential for the successful adaptation of new technologies, and collaboration and teamwork, professional conduct, and communication are all crucial aspects of this readiness.

Collaboration and teamwork are fundamental in the healthcare setting, and they become even more critical when integrating new technologies like the metaverse. The successful adaptation of such technologies often requires the involvement of multiple stakeholders, including healthcare professionals, IT specialists, administrators, and patients. Collaboration among these different groups is necessary to ensure a comprehensive and well-rounded approach to implementing and utilizing the metaverse.

By fostering collaboration and teamwork, healthcare professionals can pool their knowledge, skills, and perspectives to collectively address challenges, make informed decisions, and create effective strategies for integrating the metaverse into healthcare practice. This collaboration promotes a sense of shared responsibility and ownership, leading to a more successful and sustainable implementation of the technology.

Professional conduct is another crucial aspect of societal readiness. Healthcare professionals must adhere to ethical guidelines and professional standards when utilizing new technologies like the metaverse. This includes maintaining patient confidentiality and privacy, ensuring informed consent, and practicing within the boundaries of their expertise.

By upholding professional conduct, healthcare professionals demonstrate their commitment to providing safe, effective, and ethical care to their patients. It also instills trust and confidence in the technology and its application, both within the medical community and among patients.

Effective communication is also vital for societal readiness. Clear and open communication channels facilitate the sharing of information, ideas, and concerns among healthcare professionals, patients, and other stakeholders. It enables effective coordination, understanding, and collaboration, ensuring that everyone involved is on the same page and working towards common goals.

In the context of the metaverse, effective communication is essential for explaining the technology to patients, addressing their questions and concerns, and ensuring their comfort and engagement with its use. Clear communication also helps healthcare professionals and IT specialists to understand each other's perspectives and requirements, facilitating successful integration and utilization of the technology.

By emphasizing collaboration and teamwork, professional conduct, and effective communication, societal readiness can be achieved. This readiness fosters an environment where healthcare professionals are equipped with the skills and knowledge to utilize new technologies effectively, and patients feel confident and engaged in their use. Ultimately, this societal readiness is crucial for the successful adaptation of new technologies like the metaverse in healthcare.

**Interviewer**: Thank you for highlighting the significance of collaboration and teamwork, professional conduct, and communication as examples of societal readiness. It is evident that these aspects play a crucial role in creating an environment where new technologies can be successfully adapted and utilized, leading to improved patient care and overall healthcare outcomes.

**Interviewer**: We have our expert back with us to discuss the promising opportunities that the metaverse offers for enhancing social aspects of healthcare delivery. Thank you for joining us again. Can you elaborate on how the metaverse can bridge distances, create virtual support systems, and enable immersive training experiences to improve access, communication, and overall patient experience in healthcare?

**Participant #15**: Thank you for having me again. You're absolutely right, the metaverse offers promising opportunities to enhance social aspects of healthcare delivery. Let's explore how it can bridge distances, create virtual support systems, and enable immersive training experiences to improve access, communication, and the overall patient experience.

Firstly, the metaverse has the potential to bridge distances and overcome geographical barriers in healthcare. Through virtual platforms and immersive technologies, healthcare professionals can provide consultations, support, and even medical procedures remotely. This is particularly valuable for individuals in rural or underserved areas who may have limited access to specialized care. By leveraging the metaverse, healthcare professionals can reach patients who would otherwise face challenges in accessing necessary healthcare services.

Secondly, the metaverse can create virtual support systems for patients. It can facilitate the formation of online communities and support groups where individuals with similar health conditions or experiences can connect, share information, and provide emotional support. These virtual support systems can be particularly beneficial for patients facing rare diseases or chronic conditions, as they can find comfort, guidance, and understanding from others in similar situations. The metaverse enables a sense of belonging and community, even when physical proximity is not possible.

Additionally, the metaverse can offer immersive training experiences for healthcare professionals. Virtual reality simulations, for example, can provide realistic and interactive scenarios for training and skill development. Healthcare professionals can practice complex procedures, decision-making, and communication skills in a safe and controlled virtual environment. This immersive training enhances their competence and confidence, leading to improved patient care and outcomes.

Furthermore, the metaverse can improve communication in healthcare. It offers various tools and platforms for secure and efficient communication between healthcare professionals, patients, and other stakeholders. Virtual meetings, teleconferencing, and instant messaging enable real-time collaboration, information sharing, and decision-making, regardless of physical location. This streamlined communication enhances care coordination, reduces delays, and ensures that all relevant parties are involved in the healthcare process.

Overall, the metaverse has the potential to transform healthcare by improving access, communication, and the overall patient experience. By bridging distances, creating virtual support systems, and enabling immersive training experiences, the metaverse opens up new possibilities for delivering healthcare services and connecting individuals in ways that were previously limited by physical constraints.

However, it is important to recognize that the full potential of the metaverse in healthcare can only be realized with careful consideration of ethical and privacy concerns, as well as ongoing evaluation and adaptation of these technologies to meet the specific needs and preferences of patients and healthcare professionals.

**Interviewer**: Thank you for highlighting the promising opportunities that the metaverse offers for enhancing social aspects of healthcare delivery. It is evident that by bridging distances, creating virtual support systems, and enabling immersive training experiences, the metaverse has the potential to transform healthcare and improve access, communication, and the overall patient experience.

**Interviewer**: We have our expert back with us to discuss the importance of social readiness in effectively integrating the metaverse into healthcare. Thank you for joining us again. Can you elaborate on the significance of acceptance and readiness among patients and healthcare professionals, as well as addressing privacy and security concerns, in successfully integrating the metaverse into healthcare?

**Participant #20**: Thank you for having me again. You're absolutely right, social readiness is crucial for the effective integration of the metaverse into healthcare. Acceptance and readiness among both patients and healthcare professionals, as well as addressing privacy and security concerns, play key roles in this process.

Firstly, acceptance and readiness among patients are essential for the successful integration of the metaverse into healthcare. Patients need to be willing to embrace this technology, understand its potential benefits, and feel comfortable using it as part of their healthcare experience. Education and awareness campaigns can play a significant role in promoting patient acceptance and readiness by providing information about the metaverse, its applications in healthcare, and addressing any concerns or misconceptions.

Healthcare professionals also need to be ready and willing to utilize the metaverse in their practice. They should recognize its potential benefits, be open to learning and adapting to new technologies, and have the necessary training and support to effectively utilize the metaverse. Education and training programs can help healthcare professionals develop the skills and knowledge required to integrate the metaverse into their practice and provide high-quality care using these technologies.

Addressing privacy and security concerns is crucial for the successful integration of the metaverse into healthcare. Patients need to have confidence that their personal health information will be protected and that their privacy will be maintained in virtual environments. Healthcare professionals must ensure that appropriate safeguards, such as encryption, access controls, and secure platforms, are in place to protect patient data from potential breaches.

Regulatory frameworks and guidelines should be established to govern the use of the metaverse in healthcare and address privacy and security concerns. These frameworks should outline the responsibilities of healthcare professionals, technology providers, and patients in safeguarding patient data and maintaining confidentiality. Compliance with these guidelines is crucial to build trust and ensure the ethical and responsible use of the metaverse in healthcare.

By addressing acceptance and readiness among patients and healthcare professionals, and by addressing privacy and security concerns, the metaverse can be effectively integrated into healthcare. This integration can lead to improved access, enhanced communication, and better patient experiences. However, it is important to approach this integration with careful consideration of ethical and privacy considerations, and to continually evaluate and adapt the use of the metaverse to meet the unique needs and preferences of patients and healthcare professionals.

**Interviewer**: Thank you for emphasizing the significance of acceptance and readiness among patients and healthcare professionals, as well as addressing privacy and security concerns, in effectively integrating the metaverse into healthcare. It is evident that social readiness and addressing these concerns are crucial for the successful integration of the metaverse and to ensure the confidentiality of patient data, protect against potential breaches, and provide a safe and trusted healthcare environment.

## **Early Foundations**

**Interviewer**: Today we have an expert in the field of digital professionalism in healthcare, specifically in the context of teaching basic digital technology in the metaverse. Thank you for joining us. Can you share your thoughts on the importance of preparing lessons for teaching in the metaverse for healthcare specialists?

**Participant** **#1**: Thank you for having me. Preparing lessons for teaching in the metaverse is indeed a crucial and effective aspect of digital professionalism in healthcare. As the metaverse becomes increasingly integrated into healthcare practices, it is essential for healthcare specialists to be prepared to teach basic digital technology skills to their patients and colleagues.

Preparing lessons allows healthcare specialists to structure and deliver educational content effectively. It ensures that the information is presented in a clear and organized manner, making it easier for learners to understand and apply. By planning and preparing lessons, healthcare specialists can also identify the key concepts and skills that need to be taught, ensuring a comprehensive and targeted approach to digital technology education.

Teaching basic digital technology skills is essential because it empowers patients and colleagues to navigate the digital landscape confidently. In today's world, digital literacy is becoming increasingly important in healthcare, ranging from accessing electronic health records to utilizing telemedicine platforms. By equipping individuals with the necessary digital skills, healthcare specialists can enhance patient engagement, improve healthcare outcomes, and promote overall digital professionalism.

**Interviewer**: How can healthcare specialists effectively prepare lessons for teaching basic digital technology in the metaverse?

**Participant** **#1**: Effective preparation of lessons for teaching basic digital technology in the metaverse requires a systematic approach. Healthcare specialists should start by identifying the specific digital skills that need to be taught. This can include basic computer literacy, internet usage, and specific healthcare-related applications or platforms.

Once the skills have been identified, healthcare specialists can break down the content into manageable chunks and design lesson plans accordingly. They should consider the learning objectives, instructional strategies, and assessment methods that align with the needs and learning styles of their audience. Additionally, incorporating interactive elements, such as virtual simulations or demonstrations, can enhance the learning experience in the metaverse.

It is also important for healthcare specialists to stay updated on the latest digital technologies and trends in healthcare. This allows them to provide accurate and relevant information to their learners. They can access online resources, attend webinars or workshops, or collaborate with other healthcare professionals to ensure that their lessons reflect current best practices.

**Interviewer**: Thank you for highlighting the importance of preparing lessons for teaching basic digital technology in the metaverse for healthcare specialists. It is evident that being prepared and equipped to teach digital skills is essential for healthcare professionals to empower their patients and colleagues in navigating the digital landscape effectively.

**Interviewer**: Today we have with us an expert in the field of digital professionalism in healthcare, specifically in the context of professional development and learning in the metaverse. Thank you for joining us. Can you please share your thoughts on the importance of professional development in learning for healthcare professionals in the metaverse?

**Participant #4**: Thank you for having me. Professional development in learning is indeed a crucial and effective aspect of digital professionalism in healthcare, especially in the metaverse. As technology continues to advance and shape the healthcare landscape, it is essential for healthcare professionals to stay updated and continuously develop their skills and knowledge.

Professional development in learning enables healthcare professionals to keep up with the rapidly evolving digital technologies and their applications in healthcare. It ensures that they are equipped with the latest tools, techniques, and best practices to provide high-quality care in the metaverse. By engaging in ongoing learning, healthcare professionals can enhance their digital proficiency, stay abreast of emerging trends, and adapt to new challenges and opportunities.

In the metaverse, professional development in learning is particularly vital as it allows healthcare professionals to navigate the unique dynamics and complexities of virtual environments. They can acquire the necessary skills in virtual communication, collaboration, and patient engagement to deliver effective and patient-centered care in the digital space.

**Interviewer**: How can healthcare professionals engage in professional development in learning to stay updated in the metaverse?

**Participant #4**: Healthcare professionals can engage in various activities to promote professional development and learning in the metaverse. One effective approach is to participate in continuing education programs and courses that focus on digital technologies and their applications in healthcare. These programs can be offered online or through virtual platforms, allowing professionals to learn at their own pace and convenience.

Another valuable avenue for professional development is attending conferences, workshops, and webinars that specifically address digital professionalism in healthcare. These events provide opportunities to learn from experts in the field, share experiences, and gain insights into the latest advancements and best practices.

Healthcare professionals can also join professional organizations or communities that focus on digital healthcare and the metaverse. These platforms offer networking opportunities, access to resources, and forums for knowledge exchange and collaboration.

Additionally, staying updated through self-directed learning is crucial. Healthcare professionals can explore online resources, read academic journals, and follow reputable blogs or websites that provide insights and updates on digital technologies in healthcare.

**Interviewer**: Thank you for emphasizing the importance of professional development in learning for healthcare professionals in the metaverse. It is evident that staying updated and continuously developing skills and knowledge is essential for healthcare professionals to effectively navigate the digital landscape and provide the best possible care in virtual environments.

**Interviewer**: We have our expert back with us to discuss the importance of nurses having readiness to teach in the development or implementation of emerging technologies like the metaverse. Thank you for joining us again. Can you elaborate on the significance of training and digital professionalism in empowering healthcare specialists to embrace and adopt new technologies securely and effectively, specifically in the context of the metaverse?

**Participant #5**: Thank you for having me again. You're absolutely right, as digital technologies like the metaverse become mainstream, it is essential for nurses and healthcare specialists to have the readiness to teach and guide their peers in the development and implementation of these emerging technologies. Training and digital professionalism play crucial roles in empowering healthcare specialists to embrace and adopt new technologies securely and effectively.

Training is essential to ensure that healthcare specialists are equipped with the knowledge and skills necessary to navigate and utilize the metaverse. By providing training on basic digital technology, nurses can empower their peers to understand the functionalities and operations within the metaverse. This includes teaching them how to navigate virtual environments, understand connectivity and hardware requirements, and utilize the various features and tools available in the metaverse.

Digital professionalism is equally important. Healthcare specialists need to understand the ethical considerations, privacy concerns, and best practices when utilizing the metaverse. They should be aware of the potential risks and challenges associated with these technologies and know how to address them. Digital professionalism involves using the metaverse responsibly, respecting patient privacy, and adhering to ethical guidelines and regulatory requirements.

By being trained and embracing digital professionalism, healthcare specialists can serve as mentors and guides for their peers in the development and implementation of the metaverse. They can provide support, share their knowledge and experiences, and help their colleagues navigate the complexities of using these technologies securely and effectively.

Moreover, training and digital professionalism also enable healthcare specialists to advocate for the adoption of new technologies like the metaverse within their organizations. By demonstrating the benefits and potential impact of these technologies, healthcare specialists can influence decision-makers and create a culture that embraces innovation and technology in healthcare.

It is important to note that training and digital professionalism should be ongoing and adaptable. As technologies continue to evolve, healthcare specialists need to stay updated and continuously enhance their skills and knowledge. They should actively seek opportunities for professional development, engage in interdisciplinary collaborations, and stay informed about the latest advancements in the metaverse and other digital technologies relevant to healthcare.

In summary, training and digital professionalism are crucial in empowering healthcare specialists to embrace and adopt new technologies like the metaverse securely and effectively. By teaching basic digital technology, healthcare specialists can guide their peers, promote a culture of innovation, and ensure the responsible and ethical use of these technologies in healthcare settings.

**Interviewer**: Thank you for emphasizing the importance of training and digital professionalism in empowering healthcare specialists to embrace and adopt new technologies securely and effectively. It is evident that by providing guidance and teaching basic digital technology, healthcare specialists can play a vital role in facilitating the development and implementation of emerging technologies like the metaverse in a responsible and ethical manner.

**Interviewer**: We have our expert back with us to discuss the importance of healthcare professionals not being afraid to use new technologies and the significance of professional development in the context of the metaverse in healthcare. Thank you for joining us again. Can you elaborate on how practicing in a simulated environment encourages learning and the importance of healthcare professionals continually updating their knowledge and skills to keep pace with new advancements and technologies?

**Participant #7**: Thank you for having me again. You're absolutely right, healthcare professionals, including physicians and nurses, should not be afraid to use new technologies. Practicing in a simulated environment can indeed encourage learning and the adoption of new technologies, especially in the context of the metaverse.

Simulated environments provide a safe and controlled space for healthcare professionals to explore and familiarize themselves with new technologies like the metaverse. By practicing in these environments, healthcare professionals can gain hands-on experience, experiment with different functionalities, and develop their skills and confidence in using the technology. This type of experiential learning enables healthcare professionals to bridge the gap between theory and practice, ensuring a smoother transition to real-world applications.

Moreover, professional development and continuous learning are crucial for healthcare professionals to keep pace with new advancements and technologies. The field of healthcare is constantly evolving, and new technologies, including those in the metaverse, are being developed at a rapid pace. To provide the best possible care to patients, healthcare professionals must continually update their knowledge and skills.

Professional development programs, workshops, conferences, and online resources play a vital role in enabling healthcare professionals to stay updated. These opportunities offer them the chance to learn about new advancements, understand their implications for healthcare practice, and acquire the necessary skills to effectively utilize these technologies. By investing in professional development, healthcare professionals can ensure that they are well-prepared to embrace new technologies like the metaverse and leverage their potential benefits in patient care.

Furthermore, continuous learning and updating of knowledge and skills also contribute to digital professionalism. Digital professionalism involves understanding the ethical considerations, privacy concerns, and best practices when utilizing technologies like the metaverse. By staying updated, healthcare professionals can ensure they are aware of the latest guidelines, regulations, and ethical frameworks related to the use of these technologies.

In summary, practicing in a simulated environment encourages learning and the adoption of new technologies, while professional development and continuous learning are crucial for healthcare professionals to keep pace with advancements and technologies in the metaverse. By embracing these opportunities, healthcare professionals can enhance their skills, build confidence, and provide the best possible care to their patients. It also ensures that they are equipped with the knowledge and understanding to navigate the ethical and professional considerations associated with the use of these technologies.

**Interviewer**: Thank you for highlighting the importance of healthcare professionals not being afraid to use new technologies and the significance of professional development in the context of the metaverse in healthcare. It is evident that by practicing in simulated environments and continually updating their knowledge and skills, healthcare professionals can embrace new advancements and technologies, improve patient care, and navigate the ethical considerations associated with the use of technologies like the metaverse.

**Interviewer**: We have our expert back with us to discuss the importance of continuous learning and professional awareness in embracing new technologies like the metaverse in healthcare. Thank you for joining us again. Can you elaborate on how continuous learning enables healthcare professionals to understand the potential benefits and risks associated with the metaverse and make informed decisions in their professional practice? Additionally, how does continuous learning help healthcare professionals gain a deeper understanding of the ethical considerations, privacy concerns, and legal obligations related to the use of this technology?

**Participant #8**: Thank you for having me again. You're absolutely right, continuous learning and professional awareness are crucial in embracing new technologies like the metaverse in healthcare. Let's explore how continuous learning enables healthcare professionals to understand the potential benefits and risks associated with the metaverse and make informed decisions in their professional practice.

Continuous learning allows healthcare professionals to stay updated on the latest advancements and research related to the metaverse. This includes understanding the potential benefits that the metaverse can offer in healthcare, such as improved access, enhanced communication, and immersive training experiences. By staying informed, healthcare professionals can identify opportunities to leverage the metaverse in their practice and provide better care to their patients.

However, continuous learning also involves understanding the potential risks and challenges associated with the metaverse. Healthcare professionals need to be aware of privacy concerns, ethical considerations, and legal obligations that come with using this technology. They should understand how patient data is protected, the importance of informed consent, and the need to maintain confidentiality in virtual environments. Continuous learning helps healthcare professionals gain a deeper understanding of these aspects, enabling them to navigate the use of the metaverse responsibly and ethically.

Moreover, continuous learning enables healthcare professionals to make informed decisions in their professional practice. By staying up to date with the latest research, guidelines, and best practices, healthcare professionals can evaluate the potential benefits and risks of using the metaverse in specific healthcare contexts. They can assess whether the use of the metaverse aligns with their patients' needs, their own expertise, and the resources available to them. This informed decision-making ensures that healthcare professionals use the metaverse in a manner that maximizes its benefits while minimizing potential risks.

Continuous learning can be facilitated through various channels, including professional development programs, conferences, workshops, and online resources. Healthcare professionals can engage in interdisciplinary collaborations, participate in discussions and knowledge-sharing platforms, and seek mentorship from experts in the field. These opportunities foster a culture of continuous learning and professional awareness, enabling healthcare professionals to successfully embrace new technologies like the metaverse in their practice.

In summary, continuous learning and professional awareness are essential for healthcare professionals to understand the potential benefits and risks associated with the metaverse, make informed decisions in their professional practice, and gain a deeper understanding of the ethical considerations, privacy concerns, and legal obligations related to its use. By engaging in continuous learning, healthcare professionals can navigate the use of the metaverse responsibly and ethically, and ultimately provide better care to their patients.

**Interviewer**: Thank you for emphasizing the importance of continuous learning and professional awareness in embracing new technologies like the metaverse in healthcare. It is evident that through continuous learning, healthcare professionals can understand the potential benefits and risks associated with the metaverse, make informed decisions, and gain a deeper understanding of the ethical considerations, privacy concerns, and legal obligations related to its use. By staying informed and engaged in continuous learning, healthcare professionals can ensure the responsible and effective integration of the metaverse into their professional practice.

**Interviewer**: We have our expert back with us to discuss the importance of professional development in learning for healthcare professionals to cope with new technologies like the metaverse. Thank you for joining us again. Can you elaborate on how professional development in learning supports healthcare professionals in staying updated, acquiring necessary skills, and fostering a mindset of lifelong learning in the context of the metaverse in healthcare? How does this enable healthcare professionals to optimize the use of metaverse technology and provide quality healthcare services in a rapidly evolving digital landscape?

**Participant #10**: Thank you for having me again. You're absolutely right, professional development in learning is critical for healthcare professionals to cope with new technologies like the metaverse. Let's delve into how professional development in learning supports healthcare professionals in staying updated, acquiring necessary skills, and fostering a mindset of lifelong learning in the context of the metaverse in healthcare.

Firstly, professional development in learning enables healthcare professionals to stay updated with the latest advancements and changes in the digital landscape, including the metaverse. The metaverse is an evolving technology, and new applications and features are continuously being developed. By engaging in professional development activities, healthcare professionals can stay informed about these advancements, understand their implications for healthcare practice, and adapt their skills and knowledge accordingly.

Acquiring necessary skills is another essential aspect of professional development in learning. As the metaverse presents unique functionalities and operations, healthcare professionals need to acquire the skills to effectively utilize this technology in their practice. Professional development programs, workshops, and training opportunities provide healthcare professionals with the necessary knowledge and practical skills to navigate virtual environments, understand connectivity and hardware requirements, and utilize the various features and tools available in the metaverse.

Furthermore, professional development in learning fosters a mindset of lifelong learning among healthcare professionals. The digital landscape is rapidly evolving, and new technologies will continue to emerge. By embracing lifelong learning, healthcare professionals can adapt to these changes, continuously enhance their skills, and remain at the forefront of healthcare innovation. This mindset encourages healthcare professionals to actively seek out new learning opportunities, engage in interdisciplinary collaborations, and stay informed about the latest advancements in the metaverse and other digital technologies relevant to healthcare.

By staying updated, acquiring necessary skills, and fostering a mindset of lifelong learning, healthcare professionals can optimize the use of metaverse technology in providing quality healthcare services. They can leverage the potential benefits of the metaverse, such as improved access, enhanced communication, and immersive training experiences, to enhance patient care and outcomes. Additionally, continuous professional development ensures that healthcare professionals are equipped to navigate the ethical considerations, privacy concerns, and legal obligations associated with the use of the metaverse, ensuring responsible and ethical use of this technology.

In summary, professional development in learning is crucial for healthcare professionals to cope with new technologies like the metaverse. By staying updated, acquiring necessary skills, and fostering a mindset of lifelong learning, healthcare professionals can optimize the use of metaverse technology in providing quality healthcare services in a rapidly evolving digital landscape. Through continuous professional development, healthcare professionals can adapt to changes, enhance their skills, and ensure the responsible and effective integration of the metaverse into their practice.

**Interviewer**: Thank you for emphasizing the importance of professional development in learning for healthcare professionals to cope with new technologies like the metaverse. It is evident that by staying updated, acquiring necessary skills, and fostering a mindset of lifelong learning, healthcare professionals can optimize the use of metaverse technology and provide quality healthcare services in a rapidly evolving digital landscape. Professional development in learning plays a critical role in ensuring healthcare professionals are well-prepared to embrace new technologies and provide the best possible care to their patients.
