# Supplementary material for: Principles of digital professionalism for the metaverse in healthcare
Source: BMC Med Inform Decis Mak. 2024 Jul 22;24:201. doi: 10.1186/s12911-024-02607-y (PMC11265428; doi:10.1186/s12911-024-02607-y)
Supplement: Supplementary file 2 — Supplementary Material 2 [file 12911_2024_2607_MOESM2_ESM.docx]

## **Supplementary file 1**

*Example MAXQDA Coding for the Interview Text*

Based on the interview text provided, here is an example of how the authors could have coded the data using MAXQDA:

**Interviewer**: We have our expert back with us to discuss the importance of continuous learning and professional awareness in embracing new technologies like the metaverse in healthcare. Thank you for joining us again. Can you elaborate on how continuous learning enables healthcare professionals to understand the potential benefits and risks associated with the metaverse and make informed decisions in their professional practice? Additionally, how does continuous learning help healthcare professionals gain a deeper understanding of the ethical considerations, privacy concerns, and legal obligations related to the use of this technology?

**Participant #8**: Thank you for having me again. You're absolutely right, continuous learning and professional awareness are crucial in embracing new technologies like the metaverse in healthcare. Let's explore how continuous learning enables healthcare professionals to understand the potential benefits and risks associated with the metaverse and make informed decisions in their professional practice.

Continuous learning allows healthcare professionals to stay updated on the latest advancements and research related to the metaverse. This includes understanding the potential benefits that the metaverse can offer in healthcare, such as improved access, enhanced communication, and immersive training experiences. By staying informed, healthcare professionals can identify opportunities to leverage the metaverse in their practice and provide better care to their patients.

However, continuous learning also involves understanding the potential risks and challenges associated with the metaverse. Healthcare professionals need to be aware of privacy concerns, ethical considerations, and legal obligations that come with using this technology. They should understand how patient data is protected, the importance of informed consent, and the need to maintain confidentiality in virtual environments. Continuous learning helps healthcare professionals gain a deeper understanding of these aspects, enabling them to navigate the use of the metaverse responsibly and ethically.

Moreover, continuous learning enables healthcare professionals to make informed decisions in their professional practice. By staying up to date with the latest research, guidelines, and best practices, healthcare professionals can evaluate the potential benefits and risks of using the metaverse in specific healthcare contexts. They can assess whether the use of the metaverse aligns with their patients' needs, their own expertise, and the resources available to them. This informed decision-making ensures that healthcare professionals use the metaverse in a manner that maximizes its benefits while minimizing potential risks.

Continuous learning can be facilitated through various channels, including professional development programs, conferences, workshops, and online resources. Healthcare professionals can engage in interdisciplinary collaborations, participate in discussions and knowledge-sharing platforms, and seek mentorship from experts in the field. These opportunities foster a culture of continuous learning and professional awareness, enabling healthcare professionals to successfully embrace new technologies like the metaverse in their practice.

In summary, continuous learning and professional awareness are essential for healthcare professionals to understand the potential benefits and risks associated with the metaverse, make informed decisions in their professional practice, and gain a deeper understanding of the ethical considerations, privacy concerns, and legal obligations related to its use. By engaging in continuous learning, healthcare professionals can navigate the use of the metaverse responsibly and ethically, and ultimately provide better care to their patients.

**Code System**

The authors developed the following code system:

Continuous Learning

1.1. Staying Updated on Metaverse Advancements

1.2. Understanding Potential Benefits

1.3. Understanding Potential Risks and Challenges

Informed Decision-Making

2.1. Evaluating Metaverse Fit

2.2. Balancing Benefits and Risks

2.3. Aligning with Patient Needs and Resources

Facilitating Continuous Learning

3.1. Professional Development Programs

3.2. Conferences and Workshops

3.3. Online Resources

3.4. Interdisciplinary Collaboration

**Coding Example**

The authors would have gone through the interview transcript and applied relevant codes to the text. For example:

Participant #8: "Continuous learning allows healthcare professionals to stay updated on the latest advancements and research related to the metaverse. This includes understanding the potential benefits that the metaverse can offer in healthcare, such as improved access, enhanced communication, and immersive training experiences."

**Coded segments:**

"Continuous learning allows healthcare professionals to stay updated on the latest advancements and research related to the metaverse" - Code: 1.1. Staying Updated on Metaverse Advancements

"understanding the potential benefits that the metaverse can offer in healthcare, such as improved access, enhanced communication, and immersive training experiences" - Code: 1.2. Understanding Potential Benefits

Participant #8: "Healthcare professionals need to be aware of privacy concerns, ethical considerations, and legal obligations that come with using this technology. They should understand how patient data is protected, the importance of informed consent, and the need to maintain confidentiality in virtual environments."

**Coded segments:**

"Healthcare professionals need to be aware of privacy concerns, ethical considerations, and legal obligations that come with using this technology" - Code: 1.3. Understanding Potential Risks and Challenges

"They should understand how patient data is protected, the importance of informed consent, and the need to maintain confidentiality in virtual environments" - Code: 1.3. Understanding Potential Risks and Challenges

The authors could continue this process throughout the interview transcript, applying relevant codes and potentially adding new codes as needed. This would allow them to systematically analyze the data and identify key themes and patterns related to continuous learning and the adoption of the metaverse in healthcare.
